# Risk factors for deep venous thrombosis of lower limbs in postoperative neurosurgical patients

**DOI:** 10.12669/pjms.325.10481

**Published:** 2016

**Authors:** Qiang Li, Zongxue Yu, Xiao Chen, Jinjun Wang, Guixi Jiang

**Affiliations:** 1 Qiang Li, Vascular Surgery Department, Qingdao Hiser Medical Group, Qingdao, Shandong Province, 266033, China; 2 Zongxue Yu, Endocrinology Department, The Third People’s Hospital of Qingdao, Qingdao, Shandong Province, 266041, China; 3 Xiao Chen, Vascular Surgery Department, Qingdao Hiser Medical Group, Qingdao, Shandong Province, 266033, China; 4 Jinjun Wang, Vascular Surgery Department, Qingdao Hiser Medical Group, Qingdao, Shandong Province, 266033, China; 5 Guixi Jiang, Vascular Surgery Department, Qingdao Hiser Medical Group, Qingdao, Shandong Province, 266033, China

**Keywords:** Risk factors, Protective factors, Deep venous thrombosis (DVT), Neurosurgery

## Abstract

**Objectives:**

To detect the risk factors for deep venous thrombosis (DVT) in patients after neurosurgery.

**Methods:**

Three hundred and seventy-six patients treated in the department of neurosurgery of our hospital from February 2013 to November 2015 were reviewed retrospectively. The clinical data including age, gender, hospital stay, operation time, occupation type, hypertension, coronary heart disease, diabetes, smoking status, drinking status, postoperative exercises, malignant tumor, and postoperative hormone or dehydrating agent were collected.

**Results:**

In this study, 52 patients were included in the DVT group and 295 patients in the Non-DVT group. There was significant difference in age, hypertension, occupation type, malignant tumors, operation time, smoking status, and postoperative exercises between the two groups (p<0.05). However, there was no significant difference in gender, drinking status, coronary heart disease, diabetes, hospital stay, and postoperative hormone or dehydrating agent (p>0.05). In multivariate analysis, age, malignant tumor, hypertension were independent risk factors, while physical labour and postoperative exercises were protective factor for DVT.

**Conclusion:**

The postoperative patients with older age, malignant tumor or hypertension should be paid high attention to prevent DVT, and postoperative exercises should be selected as precautionary measures.

## INTRODUCTION

Deep vein thrombosis (DVT) of the lower limbs is a common complication in patients suffering from surgery, and pulmonary embolism (PE) is one of the most common causes of death in patients hospitalized for surgical procedures. Especially, those patients undergoing neurosurgical procedures are at high risk for perioperative DVT and PE, which have been reported in 6% to 43%^1^, even some authors stated that incidence of DVT in neurosurgical patients was as high as 18-50%, and that of PE was 0-25%.^2^ Postoperative patients in neurosurgery department need strict control of hemorrhage risk in the perioperative period, severe neurological deficits may be associated with long-term immobilization and some brain tumors are associated with state of hypercoagulability, resulting in a higher rate of DVT in neurosurgery department.

However, DVT of the lower limbs is often asymptomatic and the sensitivity for routine screening for asymptomatic DVT of lower limbs is low, and in many patients the fatal PE is usually the first clinical manifestation.^3^ In many cases, even the fatal condition may not be diagnosed correctly until the results of the autopsy are known.^4^ Also, prophylactic methods for DVT, including aspirin, low dose heparin, elastic stockings and intermittent pneumatic calf compression have been used widely after surgery, but the thromboembolic disease is still a significant postoperative complication.^5^ For these reasons, routine and systematic prophylaxis in patients at risk may be the optimal strategy to reduce the occurrence of DVT after neurosurgery. Subsequently, it is critical to analyze the risk factors of DVT in patients after neurosurgery.

Therefore, in the current study, we retrospectively reviewed the postoperative patients in neurosurgery department of our hospital to detect the risk factors for DVT of lower limbs, to help surgeons carry out the routine and systematic prophylaxis for the fatal disease.

## METHODS

Three hundred and seventy-six patients treated in the department of neurosurgery of our hospital from February 2013 to November 2015 were reviewed retrospectively. The clinical data including age, gender, hospital stay, operation time, occupation type, hypertension, coronary heart disease, diabetes, smoking status, drinking status, postoperative exercises, malignant tumor, and postoperative hormone or dehydrating agent were collected.

The diagnosis of DVT should be confirmed by auxiliary examinations, such as Doppler ultrasound, plasma D-dimer, venography, magnetic resonance imaging venography and angiography.^6^ Those who had DVT diagnosed before neurosurgery and who developed arterial thrombosis were excluded from this study.^7^ The study was approved by the ethics committee of our hospital.

Statistical analysis was performed using SPSS 21.0 (SPSS Inc., Chicago, IL, USA). The comparison of measurement data was performed by independent 2-sample t test, and enumeration data by chi-square test between the two groups. Univariate and multivariate logistic regression analysis were used to find the correlation between variables and DVT, and the multivariate logistic regression analysis was carried out to determine the independent risk factors for DVT. P < 0.05 was considered to indicate statistical significance.

## RESULTS

Three hundred and seventy-six patients were treated in the department of neurosurgery of our hospital from February 2013 to November 2015. Among the 376 patients, twenty-nine were excluded for incomplete clinical data or other reasons, and 347 patients were included in this study. In the 347 patients, 52 were diagnosed as DVT and included in DVT group, 295 were ruled out of the diagnosis of DVT and included in the Non-DVT group. The incidence of DVT was 13.8% in this study.

Among 52 patients in DVT group, DVT occurred in left lower extremity for 23 patients, right lower extremity for 14 patients and both lower extremities for 15 patients. The affected veins included common femoral vein (3 cases), superficial femoral vein (3 cases), deep femoral vein (2 cases), popliteal vein (4 cases), anterior tibial vein (2 cases), posterior tibial vein (5 cases), peroneal vein (23 cases), and calf muscle vein (36 cases).

In this study, the comparison of the clinical characteristics between the two groups are listed in Table-I. There was significant difference in age, hypertension, occupation type, malignant tumors, operation time, smoking status, and postoperative exercises between the two groups(p<0.05), but no significant difference in gender, drinking status, coronary heart disease, diabetes, hospital stay, and postoperative hormone or dehydrating agent (p>0.05, Table-I). In multivariate analysis, age, malignant tumor, hypertension were independent risk factors, while physical labour and postoperative exercises were protective factor for DVT (Table-II).

**Table-I:** The factors for deep venous thrombosis after neurosurgery.

*Factors*	*DVT*	*Non-DVT*	*P value*

	*n=52*	*n=295*	
Age (> 50 years) (n,%)	38(73.1%)	135(45.8%)	0.0003
Gender (M/F)	32/20	164/131	0.43
Hypertension (n, %)	27(51.9%)	72(24.4%)	0.0001
Occupation (physical/mental)	14/38	144/151	0.003
Coronary heart disease (n, %)	13(25%)	88(29.8%)	0.479
Diabetes (n, %)	7(13.5%)	47(15.9%)	0.65
Hospital stay(days)	26.5±4.9	18.5±5.1	0.03
Malignant tumor (n, %)	36(69.2%)	78(26.4%)	0.000
Postoperative exercises (n, %)	12(23.1%)	138(46.8%)	0.001
Operation time>3 hours (n, %)	26(50%)	102(34.6%)	0.03
Smoking (n, %)	29(55.8%)	96(32.4%)	0.001
Drinking (n, %)	17(32.7%)	78(26.4%)	0.35
Postoperative hormone (n, %)	39(75%)	198(67.1%)	0.26
Postoperative dehydrating agent (n, %)	42(80.8%)	204(69.1%)	0.08

**Table-II:** The independent risk factors for deep venous thrombosis after neurosurgery.

*Factors*	*P value*	*OR (95% CI)*
Age (> 50 years)	0.000	3.217(1.672-6.188)
Hypertension (n, %)	0.000	3.345(1.826-6.128)
Occupation (physical labour)	0.04	0.386(0.201-0.743)
Malignant tumor (n, %)	0.000	6.260(3.290-11.910)
Postoperative exercises	0.01	0.341(0.172-0.677)

## DISCUSSION

In this study, a retrospective analysis of 376 postoperative patients in neurosurgery department of our hospital was carried out to determine the risk factors of DVT. To the best of our knowledge, few studies have been published in this regard. We believe this study may help surgeons in neurosurgery department make treatment strategy and prevent the occurrence of the fatal disease correctly.

We found that mental worker presented with a higher rate of DVT than physical workers after neurosurgery. In a study of four hundred and ninety-eight patients treated surgically in department of gynecology, Zhang and colleagues found that physical labour was protective factors for DVT.^7^ In this study, we reached the same conclusion. Prolonged sitting time, independent of physical activity, has been reported as a risk factor for various negative health outcomes.^8^ In a study of 394 cases, Healy found in multivariate analysis, that prolonged work- and computer-related seated immobility was significantly associated with an increased risk of venous thromboembolism.^9^ In another case-control study, West also came to the same conclusion.^10^ These studies demonstrated the adverse effect of some occupations on the incidence of DVT. Those mental workers usually keep on sitting in their daily life, which certainly aggravates the occurrence of DVT. In addition, we found postoperative exercises were protective factor of DVT, which is also consistent with some published literatures.^11^,^12^

In a prospective study of one hundred and ninety-six patients who had neurosurgery, Guo detected the factors including the presence of a tumor, an age greater than 50 years, hypertension, postoperative dehydration and immobility are independent risk factors of DVT.^6^ In our study, we had the similar conclusion and also found in multivariate analysis that age, malignant tumor, hypertension were independent risk factors of DVT. Patients with older age are often associated with vascular sclerosis, high blood viscosity and poor venous valve function, this lead to a high rate of lower limb DVT when combined with reduced activity postoperatively.^13^ The influence of malignant tumor and hypertention on DVT was also confirmed by some clinical studies.^14^-^16^ Although these patients may come from different departments and undergo different surgeries, we believe that the mechanism of DVT is similar.

However, different from Guo’s study, in terms of the use of dehydration agent, we found there was no significant difference between the control group and DVT group, and we suggest postoperative dehydration isn’t the independent risk factors of DVT after neurosurgery. The difference between studies may be attributed to different patients in the two studies. Moreover, in neurosurgery department dehydration agents are usually used for most of patients, which lead to difficulties in identifying it as risk factor, and this may also influence the final results between studies.

To summarize, we have found in this study that age, malignant tumor, hypertension were independent risk factors, while physical labour and postoperative exercises were protective factor for DVT.

### Limitations of the study

First, the study was performed retrospectively, while a prospective study may be better in deciding the risk factors of DVT related to the postoperative patients. Second, we concluded from this study that age, malignant tumor and hypertension were independent risk factors of DVT, but some other factors, such as perioperative bleeding, may also affect the occurrence of DVT after neurosurgery,^17^ while these factors were not studied in the current study. Third, in terms of some risk factors, our conclusion is consistent with some previous studies, but in some other factors the opinions in the study are different, it is critical to carry out more studies to clarify the above issues. Hence, we suggest a multi-centers, large scaled, clinical study to be performed in the future.
